# Circulating extrachromosomal circular DNA as a prognostic biomarker for colorectal cancer

**DOI:** 10.1186/s12964-026-02721-6

**Published:** 2026-02-07

**Authors:** Xuanmei Luo, Jian Cui, Jinxin Shi, Gaoyuan Sun, Lili Zhang, Yayu Li, Yingyu Guo, Lu Kuai, Tianhan Sun, Qi Luo, Jiahui Cai, Qi An, Wei Zhang, Fei Xiao, Gang Zhao

**Affiliations:** 1 Clinical Biobank, Beijing Hospital, National Center of Gerontology, Institute of Geriatric Medicine, Chinese Academy of Medical Sciences, No.1 Dahua Road, Dongcheng District, Beijing, 100730 China; 2 Peking University Fifth School of Clinical Medicine, Beijing Hospital, National Center of Gerontology, Beijing, China; 3 Department of General Surgery, Beijing Hospital, National Center of Gerontology, Institute of Geriatric Medicine, Chinese Academy of Medical Sciences, No.1 Dahua Road, Dongcheng District, Beijing, 100730 China; 4 Department of Pathology, Beijing Hospital, National Center of Gerontology, Institute of Geriatric Medicine, Chinese Academy of Medical Sciences, No.1 Dahua Road, Dongcheng District, Beijing, 100730 China

**Keywords:** Colorectal cancer, Extrachromosomal circular DNA, Recurrence, Mortality, Liquid biopsy

## Abstract

**Background:**

Delayed detection of recurrence significantly contributes to colorectal cancer (CRC) mortality, underscoring the need for robust prognostic biomarkers. Although extrachromosomal circular DNA (eccDNA) is a known oncogenic driver, its prognostic utility in CRC remains largely unexplored.

**Methods:**

In this 6-year prospective cohort study, full-length eccDNA profiling of 153 plasma samples was performed using Nanopore sequencing. Differential eccDNA signatures between recurrence (R, *n* = 20) and non-recurrence (NR, *n* = 133) patients enabled construction of predictive models for recurrence and mortality. Functional validation of eccDNAs was conducted in HCT116 cells.

**Results:**

Compared to NR patients, R patients exhibited enrichment of eccDNAs derived from chromosome 9, shorter median eccDNA lengths, and reduced variability in eccDNA length. All 4.9–5.0 kb eccDNAs derived from *CKM*, while other eccDNAs showed a strong genomic distribution correlation between groups (Spearman’s ρ = 0.73). Promoter-derived eccDNAs were enriched in R patients, particularly from the promoter of *CARD9* (eccPromoter-CARD9, 10.4-fold increase). Overexpression of eccPromoter-CARD9 significantly promoted CRC cell proliferation and migration. R patients exhibited elevated eccDNAs harboring the hsa-mir-374c cluster in plasma and tissues, and their corresponding miRNAs demonstrated exceptional diagnostic accuracy in CRC-related TCGA cohorts. An eccDNA-based random forest classifier achieved superior recurrence prediction accuracy (AUC > 0.8), correlating with shorter time-to-recurrence (HR = 3.79) and elevated CA125 and CEA levels. Additional eccDNA-based models effectively predicted recurrence-associated mortality (AUC ≥ 0.93).

**Conclusions:**

The plasma eccDNA landscape may serve as an early and powerful non-invasive biomarker for CRC prognostication, optimizing risk stratification and guiding personalized treatment.

**Supplementary Information:**

The online version contains supplementary material available at 10.1186/s12964-026-02721-6.

## Background

Despite advances in prevention and treatment, colorectal cancer (CRC) remains a leading global health burden, ranking as the third most common cause of cancer-related deaths [1–3]. A substantial proportion of patients with stage II/III CRC experience recurrence even after curative resection, highlighting the limitations of current histopathological Tumor Node Metastasis (TNM) staging in accurately stratifying patient risk [4]. Improved prognostic tools are urgently needed, particularly in stage II CRC, where the clinical benefit of adjuvant therapy remains controversial [5, 6]. 

Compared to conventional histopathological methods, liquid biopsy offers a non-invasive approach for real-time surveillance of recurrence risk [7, 8]. However, current biomarkers have significant limitations. Serum carcinoembryonic antigen (CEA) lacks specificity, as levels can be elevated in non-malignant conditions such as inflammatory bowel disease [9]. Although circulating tumor DNA (ctDNA) is a promising marker for recurrence, its detection is technically challenging due to its extremely low abundance in plasma (typically < 0.1% of total circulating cell-free DNA) [10]. 

Extrachromosomal circular DNA (eccDNA) is widely present in various tumor tissues and body fluids [11, 12], and it plays key roles in genomic plasticity [13], tumorigenesis [14, 15], and therapeutic resistance [16]. Therefore, circulating eccDNA molecules are by their nature functional biomarkers [17]. The covalently closed circular structure of eccDNA renders it more stable than linear ctDNA, protecting it from exonuclease degradation and mechanical shearing. Furthermore, its circular topology enables efficient signal amplification via rolling circle amplification (RCA), allowing for ultrasensitive detection.

Despite these advantages, the clinical potential of circulating eccDNA as a prognostic biomarker in CRC remains largely unexplored. To address this gap, we conducted full-length eccDNA sequencing on plasma samples from a 6-year prospective CRC cohort. This study aimed to systematically characterize circulating eccDNA features associated with CRC recurrence and mortality, and to assess their utility as non-invasive prognostic indicators.

## Methods

### Patients, study design, and clinical specimens

A total of 153 CRC participants who underwent radical surgery at Beijing Hospital were enrolled in this 6-year prospective study (Supplementary Tables 1–2). Among them, 20 patients who developed postoperative recurrence within six years were classified as the recurrence group (R group), while 133 patients without evidence of recurrence comprised the non-recurrence group (NR group). None of the patients had documented exposure to chemical agents known to cause DNA damage. Preoperative plasma samples were collected within two weeks before surgery and stored at -80 °C.

### EccDNA extraction and sequencing

Cell-free DNA was isolated from 0.3 mL of plasma using a Magnetic Serum/Plasma DNA Kit (TIANGEN). To enrich eccDNAs, Cell-free DNA was digested with 10 U of exonuclease III and 5 U of lambda exonuclease (all NEB) at 37 °C for 2 h. EccDNA was purified using SPRI beads (Beckman Coulter), amplified using phi29 DNA polymerase (NEB) at 30 °C for 60 h, and debranched using T7 endonuclease I (NEB). Sequencing libraries were constructed using a Ligation Sequencing Kit and sequenced on the PromethION platform (all Oxford Nanopore Technology). EccDNA identification was performed using eccDNA_RCA_nanopore software, with positive calls requiring ≥ 2 tandem repeats to confirm circularity [18]. 

### EccDNA annotation and analysis

To normalize for sequencing depth, raw eccDNA counts were adjusted relative to the total number of *hg38*-mapped reads. Genomic coverage of eccDNAs was computed using bedtools (v2.30.0) and visualized with the RIdeogram package (v0.2.2). Genomic element annotation was performed using HOMER (v4.11). KEGG pathway enrichment analysis was conducted via the DAVID online tool. Based on the protein-coding annotations downloaded from Ensembl BioMart, eccDNA carrying the intact gene was annotated. miRNA cluster annotations were identified using TAM (v2.0). Expression profiles and receiver operating characteristic (ROC) curves of corresponding miRNAs in TCGA datasets were analyzed using the CancerMIRNome online platform [19]. 

### Tissue eccDNA quantification and validation

Genomic DNA was extracted from tissue samples using a Magnetic Universal Genomic DNA Kit (TIANGEN). For eccDNA enrichment, 5 µg of genomic DNA was digested with 30 U of exonuclease III and 15 U of lambda exonuclease at 37 °C for 7 days. Outward-directed primers targeting eccDNA junctions were designed using Primer3web (v4.0). Quantitative PCR (qPCR) was performed to quantify eccDNA abundance using outward-directed primers and ChamQ Universal SYBR qPCR Master Mix (Vazyme). Junctional sequences were verified via Sanger sequencing.

### Diagnostic model construction

Random forest classifiers were developed using the randomForest package (v4.6-14). ROC curves were generated with the pROC package (v1.18.4). Survival analyses were conducted using the survival package (v0.4.9). Kaplan–Meier plots were used to visualize recurrence-free survival, and differences between groups were assessed using the log-rank test.

### EccDNA synthesis via GALA method

Following the GALA method [20], a truncated *CARD9* promoter region (chr9:136,374,457 − 136,374,791) was amplified from human genomic DNA using GoldenStar T6 Super PCR Mix Ver.2 (Tsingke). As a control, a 500-bp random DNA sequence with 50% GC content was generated using the “Random DNA sequence generator” software and synthesized by BGI. For eccDNA assembly, overlapping PCR fragments with complementary ends were generated and assembled using NEBuilder HiFi DNA Assembly Master Mix (NEB) at 55 °C for 60 min. The resulting product was used as template for amplifying two split-reversed complementary linear fragments, which were mixed in equimolar ratios and ligated using Taq DNA Ligase (NEB) under the following conditions: 95 °C for 5 min; 10 cycles of 95 °C for 20 seconds, 4 °C for 1 min, and 45 °C for 20 minutes. Residual linear DNA was digested with 20 U of exonuclease III (NEB) and 10 U of lambda exonuclease (NEB) at 37 °C for 1 h, followed by purification using a TIANgel Purification Kit (TIANGEN). Circularity and junctional sequences were confirmed by Sanger sequencing. All primer sequences are listed in Supplementary Table 2.

### Cell proliferation, apoptosis, and migration assays

HCT116 cells (ATCC) were cultured in Dulbecco’s Modified Eagle Medium (Meilunbio) supplemented with 10% fetal bovine serum (HyClone) at 37 °C in a 5% CO_2_ humidified incubator. eccDNAs were transfected using Lipo8000 (Beyotime). Cell viability was assessed using the Cell Counting Kit-8 (Beyotime), and absorbance was measured by a Gen5 Microplate Reader (BioTek). Migration was evaluated via wound healing assays, and wound closure was quantified using ImageJ software (NIH).

### RNA-seq and RT-qPCR validation

Total RNA was extracted with TRIzol reagent (TIANGEN), and mRNA was purified using a Dynabeads™ mRNA Purification Kit (Invitrogen). Sequencing libraries were prepared following the Illumina sample preparation protocol and sequenced as 2 × 150 bp paired-end reads. Clean reads were aligned and assembled using STAR (v2.5.4b), and differential expression analysis was performed using edgeR (v3.32.1).

For validation, total RNA was reverse transcribed using an Evo M-MLV RT premix for qPCR Kit (AGbio). qPCR was conducted with ChamQ Universal SYBR qPCR Master Mix (Vazyme), and relative expression was calculated using the 2^−ΔΔCt^ method with *ACTB* as the endogenous control. All primer sequences are provided in Supplementary Table 2.

### Statistical analysis

All statistical analyses were performed using R software (V4.0.5). Two-sided *P*-values < 0.05 were considered statistically significant. Correlations between non-normally distributed variables were assessed using Spearman’s rank correlation.

## Results

### Circulating eccDNA profiles associated with CRC recurrence

**Fig. 1:**
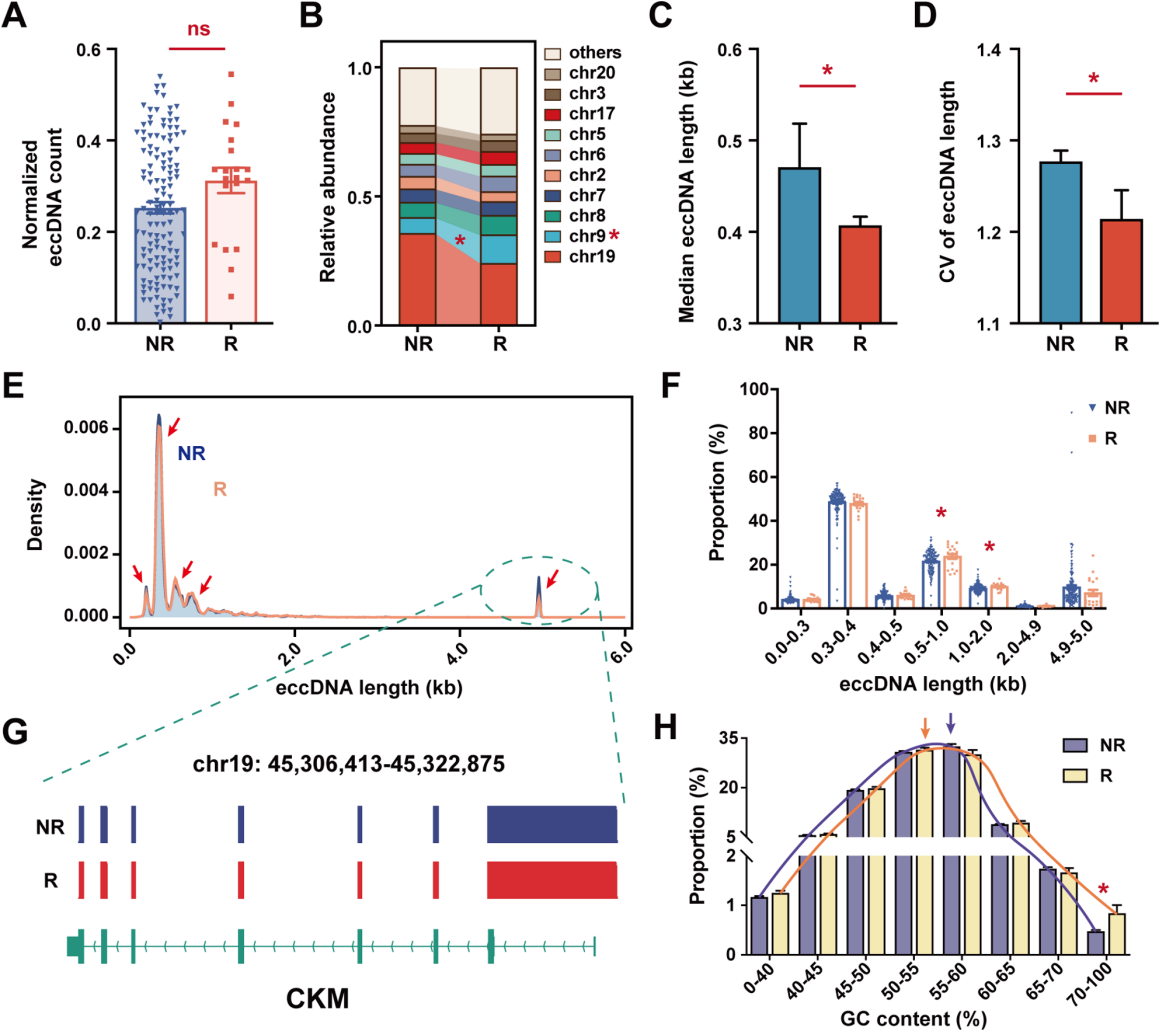
Circulating eccDNA profiles associated with CRC recurrence. **A** The normalized eccDNA count. **B** The top 10 eccDNA-enriched chromosomes. **C** The median length of eccDNAs. **D** The coefficient of variation (CV) value of eccDNA length. **E** The eccDNA length distribution. **F** Proportional abundance of eccDNAs across defined size intervals. **G** Representative genome browser tracks showing 4.9–5.0 kb eccDNAs mapping to the CKM gene. **H** The Guanine-cytosine (GC) content distribution of eccDNAs. R group, recurrence group; NR group, non-recurrence group. *, *P* < 0.05. Data are presented as mean ± SEM

In this prospective cohort of 153 CRC patients, 20 individuals (13.1%) experienced tumor recurrence during the 6-year follow-up period (Supplementary Tables 1–2). Normalized plasma eccDNA counts showed no significant difference between the recurrence (R) and non-recurrence (NR) groups (Fig. 1A). The top 10 chromosomal distribution analysis revealed that the highest proportion of circulating eccDNAs derived from chromosome (chr) 19 in both NR (35.82 ± 2.42%) and R (24.14 ± 4.95%) groups However, eccDNAs from chr9 were significantly enriched in the R group (Fig. 1B).

We then characterized the size profile of circulating eccDNAs. R patients exhibited significantly shorter median eccDNA lengths and reduced length variability, suggesting a more uniform eccDNA biogenesis process in recurrent tumors (Fig. 1C-D). Both groups showed five major peaks in length distribution (Fig. 1E), yet eccDNAs within 0.5–1.0 kb and 1.0–2.0 kb size ranges were significantly more abundant in the R group (Fig. 1F). Remarkably, all eccDNAs within the 4.9–5.0 kb size range mapped to the *CKM* gene locus, spanning its entire coding region and the first intron (Fig. 1G).

The peak guanine–cytosine (GC) content of circulating eccDNAs differed slightly between groups (R: 50–55% vs. NR: 55–60%), while eccDNAs with ultra-high GC content (70–100%) were significantly enriched in the R group (Fig. 1H).

### Preferential selection of promoter elements in recurrence-associated eccDNAs

**Fig. 2:**
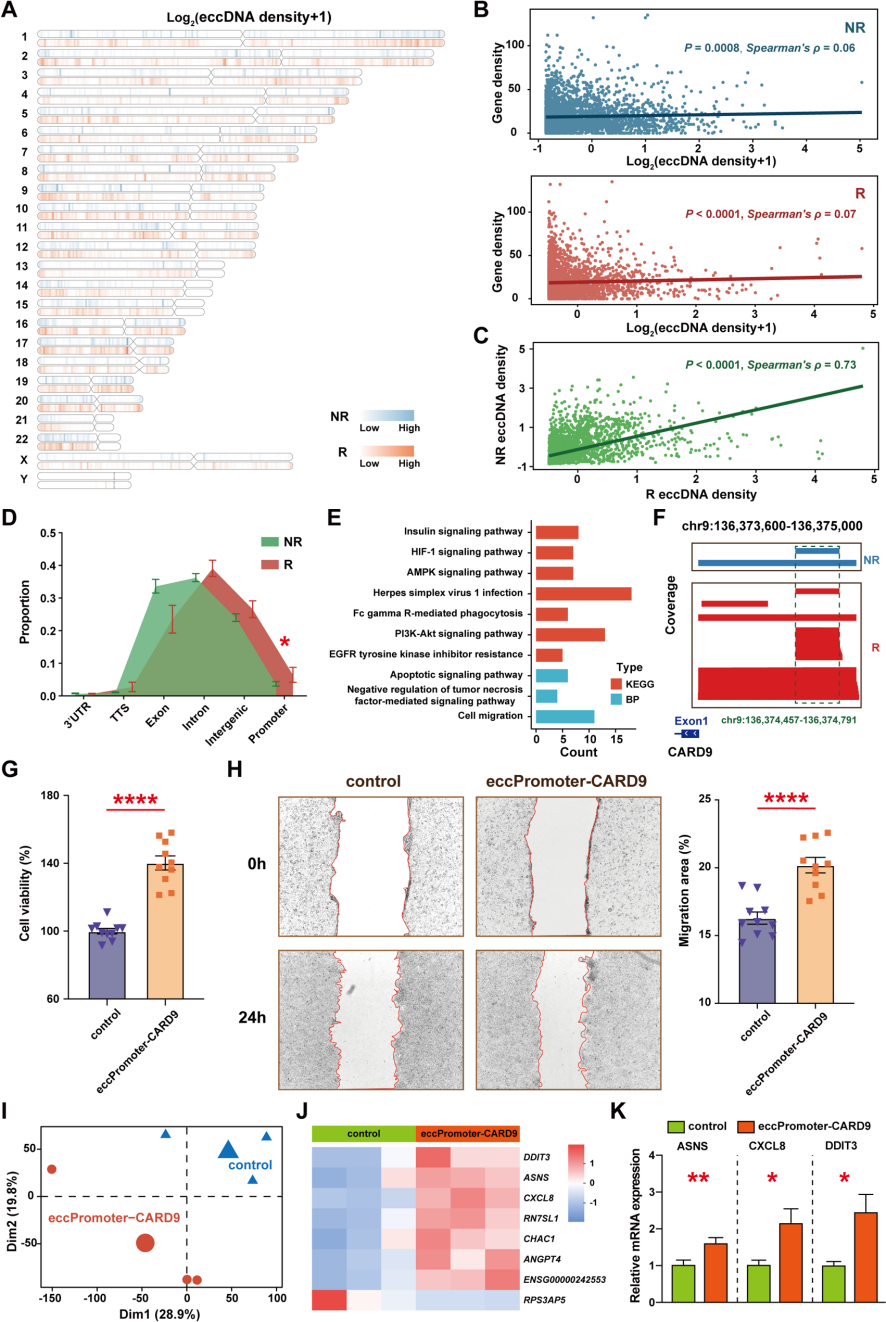
Genomic distribution and annotation of circulating eccDNAs in recurrence (R) and non-recurrence (NR) groups. **A** Genome-wide distribution profile of eccDNAs. **B** Spearman correlation between eccDNA density and genomic gene density. **C** Spearman correlation of eccDNA density patterns between R and NR groups. **D** Genomic element annotation of eccDNAs. 3’ UTR, 3’ untranslated region; TTS, transcription termination site. **E** Functional enrichment analysis (KEGG pathways and BP terms) of genes associated with elevated eccPromoter counts in the R group. **F** Representative browser tracks showing eccDNA coverage at the *CARD9* promoter locus in R and NR groups. **G** Cell viability assay detected by CCK-8 kit of eccPromoter-CARD9 in HCT116 cells. **H** Cell migration assay detected by wound healing test of eccPromoter-CARD9 in HCT116 cells. **I** Principal component analysis of transcriptomic profiles following eccPromoter-CARD9 treatment. **J** Expression heatmap of differentially expressed genes between R and NR groups. **K** Validation of gene expressions by RT-qPCR assay. *, *P* < 0.05; **, *P* < 0.01; ****, *P* < 0.0001. Data are presented as mean ± SEM

To explore the functional origins of circulating eccDNAs, we annotated their genomic features. Apart from the consistent presence of 4.9–5.0 kb eccDNAs derived from the *CKM* locus, most eccDNAs originated from randomly distributed genomic regions (Fig. 2A). Although eccDNA density was only weakly correlated with gene density in both groups (Fig. 2B), strong correlation was observed between the eccDNA distributions of R and NR groups (Fig. 2C).

Approximately 90% of eccDNAs originated from exonic, intronic, and intergenic regions (Fig. 2D). However, promoter-derived eccDNAs (eccPromoters) were significantly enriched in the R group. Further analysis identified 717 genes associated with elevated eccPromoter counts in R patients compared to NR controls (|log_2_FC| > 1 and FDR < 0.05). Gene Ontology enrichment analysis linked these genes to cell migration and apoptosis (Fig. 2E). KEGG analysis further implicated their involvement in key immune-related and oncogenic pathways, including HIF-1 signaling pathway [21], EGFR tyrosine kinase inhibitor resistance, and Herpes simplex virus 1 infection [22]. 

Consistent with prior observations across multiple cancer types [23–25], our analysis confirmed the presence of *MYC*-harboring eccDNAs in CRC patients. However, unlike previous studies that employed DNA fragment assembly algorithms to characterize large-sized *MYC*-harboring eccDNAs, our approach—which did not incorporate such assembly strategies—specifically enabled the detection of relatively small eccDNAs carrying the *MYC* promoter region (chr8:127,735,862–127,736,189). Subsequent quantitative analysis demonstrated that the abundance of these *MYC* promoter-harboring eccDNAs did not differ significantly between R and NR patients. These findings suggest that while *MYC*-associated eccDNAs may contribute to CRC pathogenesis, their presence lacks prognostic value for predicting tumor recurrence.

Among all differentially enriched eccPromoters between the two groups, those carrying the promoter of *CARD9* (eccPromoter-CARD9) exhibited the greatest difference, exhibiting a 10.4-fold enrichment in the R group (FDR < 0.0001). eccPromoter-CARD9 was detected in 5 of 20 R patients (25%) but only 2 of 133 NR patients (1.52%) (Fig. 2F). We selected the eccPromoter-CARD9 sequence (chr9:136,374,457–136,374,791), which was most frequently observed in these samples, to synthesize eccDNA molecules and assess their oncogenic potential. eccPromoter-CARD9 significantly enhanced key oncogenic phenotypes in the human CRC cell line HCT116, increasing cell viability by 140.2 ± 4.1% and enhancing migration area to 20.2 ± 1.8%, compared to 16.3 ± 1.4% in controls (Fig. 2G-H).

To characterize the transcriptional effects of eccPromoter-CARD9, we performed RNA-seq analysis. Principal component analysis revealed distinct gene expression profiles between eccPromoter-CARD9-transfected and control cells (Fig. 2I). Eight genes were differentially expressed (|log_2_FC| > 1 and FDR < 0.05), with seven upregulated and one downregulated (Fig. 2J). RT-qPCR analysis validated the upregulation of *DDIT3 (DNA Damage Inducible Transcript 3)*, *ASNS (Asparagine Synthetase)*, and *CXCL8 (C-X-C Motif Chemokine Ligand 8)* (Fig. 2K). These genes have established roles in poor CRC prognosis [26–28]. KEGG enrichment analysis linked these genes to “transcriptional misregulation in cancer”, “non-alcoholic fatty liver disease”, and “lipid and atherosclerosis”. Together, these results established eccPromoter-CARD9 as a strong recurrence-linked element in CRC, supporting its dual potential as both a prognostic biomarker and a putative oncogenic driver.

### Enrichment of eccDNA carrying the hsa-mir-374c cluster in R patients

**Fig. 3:**
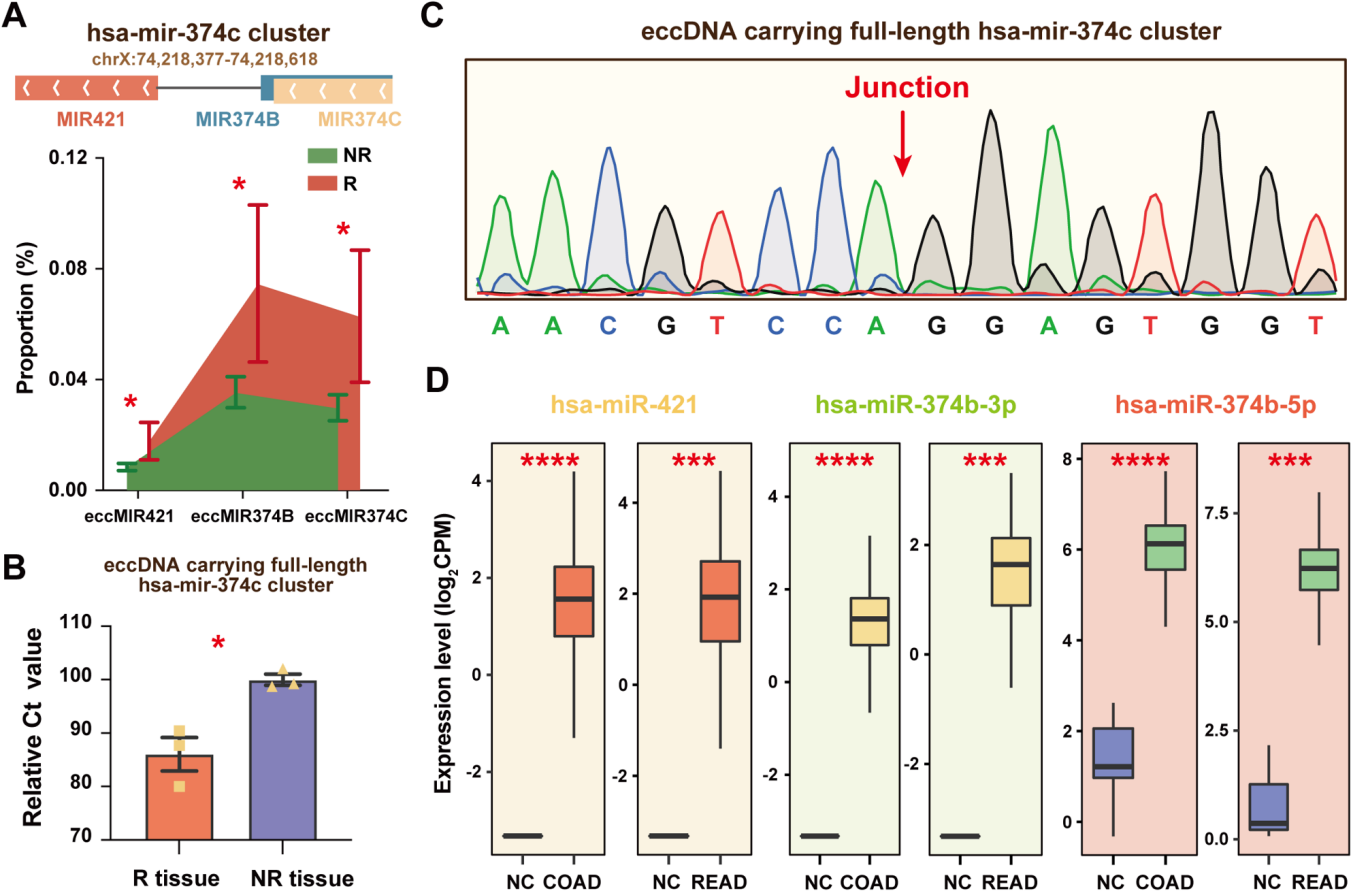
The abundance of eccDNAs carrying the hsa-mir-374c cluster and their corresponding mature miRNA expressions. **A** Genomic localization (*hg38*) of the hsa-mir-374c cluster and the proportion of eccDNAs carrying its components in recurrence (R) and non-recurrence (NR) groups. **B**-**C** The abundance of eccDNAs carrying the full-length hsa-mir-374c cluster in CRC tissues of R and NR groups and the validation of junctional sequences using Sanger sequencing. **D** Expression levels of hsa-miR-421, hsa-miR-374b-3p, and hsa-miR-374b-5p in COAD and READ cohorts. *, *P* < 0.05; ***, *P* < 0.001; ****, *P* < 0.0001. Data are presented as mean ± SEM

eccDNAs carrying full-length miRNAs can be transcribed into mature miRNA molecules [29]. In R patients, eccDNAs carrying full-length MIR421, MIR374B, or MIR374C sequences were significantly elevated compared to NR controls (Fig. 3A). These three miRNAs comprised the hsa-mir-374c cluster in the human genome (Fig. 3A). The overall abundance of eccDNAs carrying the full-length hsa-mir-374c cluster was significantly higher in R patients (0.018 ± 0.007%) than in NR patients (0.008 ± 0.001%; *P* < 0.05). This enrichment was also observed in CRC tissue samples from R patients (*n* = 3) compared to NR controls (*n* = 3) (Fig. 3B). Sanger sequencing confirmed that these eccDNAs had consistent junctional sites across all CRC tissues (Fig. 3C).

Three mature miRNAs produced by the hsa-mir-374c cluster—hsa-miR-421, hsa-miR-374b-3p, and hsa-miR-374b-5p—were significantly overexpressed in tumor tissues from two CRC-related TCGA cohorts: colon adenocarcinoma (COAD) and rectum adenocarcinoma (READ) (Fig. 3D). We found that these individual miRNAs demonstrated excellent diagnostic performance for distinguishing CRC tumors from normal tissues. In COAD, their AUCs were 0.95 (95% confidence interval [CI]: 0.91–0.99), 0.98 (95% CI: 0.97–0.98), and 1.00 (95% CI: 1.00–1.00), respectively. Similar performance was observed in READ, with AUCs of 0.98 (95% CI: 0.96–0.99), 0.98 (95% CI: 0.97–1.00), and 1.00 (95% CI: 1.00–1.00), respectively. Although circulating miRNAs achieve relative stability through vesicular encapsulation or protein binding, RNA molecules in general remain highly susceptible to degradation by ubiquitous RNA hydrolases and metal ions. This intrinsic instability, which is particularly pronounced during RNA extraction, renders RNA less stable than DNA and poses a significant challenge to its clinical translation as a biomarker [30, 31]. 

### Circulating eccDNA panel as a novel prognostic biomarker for CRC recurrence

To evaluate the clinical utility of circulating eccDNAs in stratifying recurrence risk in CRC patients, we constructed a random forest machine learning model using eccDNAs harboring gene regions (exon, intron, 3’ untranslated region [3’ UTR], transcription termination site [TTS], or promoter), hereafter referred to as eccGenes. A total of 153 CRC patients (Supplementary Table 1) were randomly allocated to training (*n* = 92) and test (*n* = 61) cohorts at an approximate ratio of 6:4 using recurrence status-stratified randomization. This approach ensured no overlap between the two subsets while maintaining balanced recurrence prevalence between the training set (15.0%, 12 R/80 NR) and test set (15.1%, 8 R/53 NR). The stratified random assignment thus supported independent and unbiased validation of the prognostic model.

**Fig. 4:**
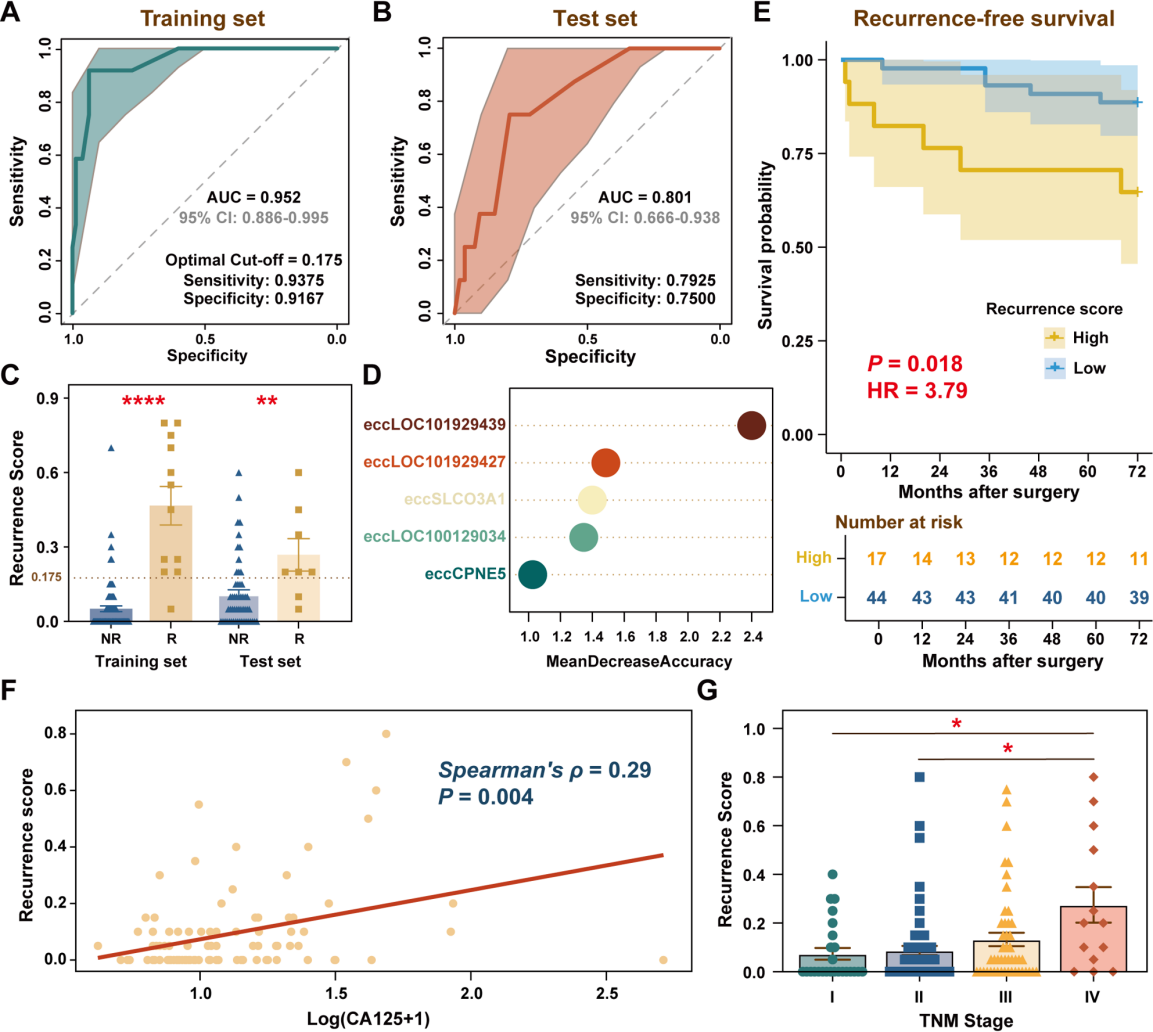
Development and validation of an eccGene-based predictive model for colorectal cancer recurrence. **A**-**B** Receiver operating characteristic curves of a random forest model in the (**A**) training set and (**B**) test set. The optimal cut-off score (0.175) was determined from the training set. **C** The distribution of recurrence scores generated by the random forest model across both cohorts. **D** The top 5 eccGene biomarkers ranked by mean decrease in accuracy score. **E** Kaplan–Meier analysis of recurrence-free survival for high- and low-risk groups stratified by the optimal cut-off score (derived from the training set via Youden’s index) in the test set. **F** Spearman correlation between recurrence scores and serum levels of cancer antigen 125 (CA125). **G** Recurrence scores in different Tumor Node Metastasis (TNM) stages (I, II, III, and IV). 95% CI, 95% confidence interval; AUC, area under the receiver operating characteristic curve; HR, hazard ratio. *, *P* < 0.05; **, *P* < 0.01; ****, *P* < 0.0001. Data are presented as mean ± SEM

Using HOMER-based annotations, we identified 13 significant eccGenes (AUC > 0.68) in the training cohort and selected them as input features for model training. The resulting model achieved an AUC of 0.952 (95% CI: 0.886–0.995) in the training set (Fig. 4A). Based on Youden’s index, an optimal cut-off score of 0.175 was established, yielding high prognostic accuracy with a sensitivity of 0.9375 and specificity of 0.9167. When applied to the test set, the model retained robust performance, with an AUC of 0.801 for distinguishing R from NR patients (Fig. 4B). Moreover, predicted recurrence scores were significantly higher in R patients than NR patients in both datasets (Fig. 4C). The top 5 most informative eccGenes, as ranked by feature importance in the random forest model, are shown in Fig. 4D. Among the five top-ranked eccGene biomarkers, copy-number alterations of two protein-coding genes (*CPNE5* and *SLCO3A1*) were previously identified through whole-exome sequencing analysis from 1,015 CRC patients [32]. The remaining three biomarkers, which are uncharacterized long non-coding RNAs, were not accessed for copy-number alterations in the cBioPortal analysis, as they fall outside the scope of conventional protein-centric alteration profiling [33]. 

We further stratified patients in the test set into high- and low-risk groups based on the optimal cut-off score determined by the Youden’s index in the training set. Kaplan–Meier analysis revealed significantly reduced recurrence-free survival in the high-risk group (Fig. 4E), with a markedly increased recurrence risk (HR = 3.79; 95% CI: 1.16–12.43; *P* < 0.05).

To contextualize the prognostic performance of our recurrence score model against established clinical biomarkers, we assessed the correlation with preoperative serum levels of three conventional tumor markers—cancer antigen 125 (CA125), carbohydrate antigen 19 − 9 (CA19-9), and CEA—each of which has been previously associated with adverse outcomes in CRC [34–36]. Spearman correlation analysis demonstrated a statistically significant positive association between the recurrence scores and CA125 (*ρ* = 0.29, *P* = 0.004; Fig. 4F) as well as CEA (*ρ* = 0.20, *P* = 0.015; Supplementary Fig. 1). In contrast, no significant correlation was observed between the recurrence score and CA19-9 (*P* > 0.05). These results not only underscore the clinical relevance of our recurrence score but also suggest its alignment with established markers of tumor burden and biological aggression, thereby supporting its utility in postoperative risk stratification. Additionally, stratification of all patients by TNM stage demonstrated a progressive increase in recurrence scores with advancing stage (Fig. 4G). Notably, scores were significantly higher in stage IV patients compared to those with early-stage disease (TNM I/II), underscoring the potential of the recurrence score in clinical risk stratification.

### Circulating eccDNA panel as a novel prognostic biomarker for CRC-related death

**Fig. 5:**
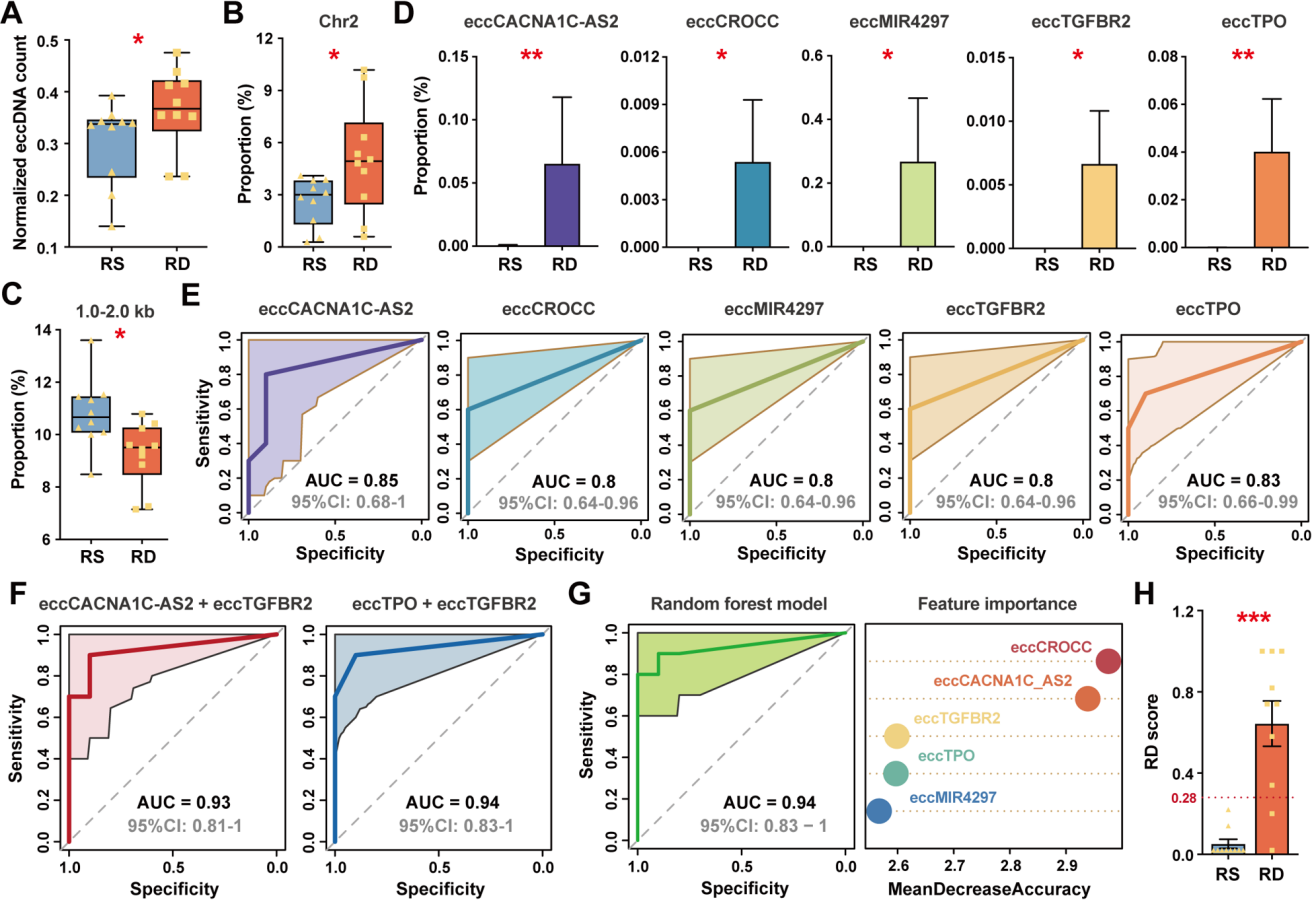
EccGene-based models for predicting recurrence-associated death in colorectal cancer. **A** Comparison of normalized eccDNA abundance between recurrence-associated survivor (RS) and recurrence-associated death (RD) groups. **B** The proportion of chromosome (chr) 2-derived eccDNAs. **C** The proportion of eccDNAs within 1.0–2.0 kb. **D** The proportion of five differential eccGenes between RS and RD groups (all P < 0.05). **E** Receiver operating characteristic curves of these five eccGenes (all AUCs ≥ 0.8). **F** Receiver operating characteristic curves of two differential eccGenes combinations (all AUCs ≥ 0.9). **G** Performance evaluation and feature importance ranking of the random forest model incorporating these five eccGenes. **H** Distribution of RD scores. The optimal cut-off score (0.28) was determined based on Youden’s index. 95%CI, 95% confidence interval; AUC, area under the receiver operating characteristic curve. *, *P* < 0.05; **, *P* < 0.01; ***, *P* < 0.001. Data are presented as mean ± SEM

Early prediction of cancer deaths allows for more timely and precise treatment interventions, potentially improving clinical outcomes. Building on the prognostic potential of eccDNAs for recurrence, we next investigated their relevance in predicting recurrence-associated deaths. We analyzed 20 recurrent CRC patients, stratified according to whether recurrence-associated death (RD) occurred (*n* = 10) or whether they had survived at study endpoint six years post-surgery (RS; *n* = 10). RD patients exhibited a significantly greater eccDNA burden compared to RS patients, with notable enrichment in eccDNAs originating from chr2 (Fig. 5A-B). Size profiling revealed a significantly reduced proportion of eccDNAs within 1000–2000 bp in RD patients (Fig. 5C).

Distinct differences in eccGene composition were also observed between groups. Five eccGenes—eccCACNA1C-AS2, eccCROCC, eccMIR4297, eccTGFBR2, and eccTPO—were significantly more abundant in RD patients (Fig. 5D). Individually, each of these biomarkers demonstrated strong discriminatory ability, with AUC values ranging from 0.80 to 0.85 (Fig. 5E). Notably, combining biomarkers enhanced predictive performance, particularly eccCACNA1C-AS2 + eccTGFBR2 (AUC = 0.93, 95% CI:0.81-1.00) and eccTPO + eccTGFBR2 (AUC = 0.94, 95% CI:0.83-1.00; Fig. 5F).

Subsequently, we developed a random forest classifier incorporating all five eccGenes, which achieved excellent predictive accuracy for recurrence-related deaths, with an AUC of 0.94 (95% CI: 0.83–1.00; Fig. 5G). The model-derived RD scores were significantly elevated in RD patients, with an optimal cut-off of 0.28 (Youden’s index), achieving a sensitivity of 0.80 and a specificity of 1.00 (Fig. 5H).

## Discussion

Growing evidence highlights the pivotal role of eccDNAs in tumorigenesis and neoplastic progression across various malignancies [37–39]. Recently, eccDNA has also gained recognition as a promising diagnostic biomarker [40, 41]. However, its association with clinical outcomes, particularly recurrence and disease-specific mortality in CRC, remains poorly defined. In this study, we revealed significant aberrations in the circulating eccDNA landscape of recurrent CRC patients, underscoring its potential as a novel, non-invasive prognostic biomarker.

Recurrence remains a leading cause of poor outcomes. Current liquid biopsy tools to monitor disease recurrence face limitations: ctDNA [42] is confounded by non-tumor-derived background noise, microbiome DNA approaches [43, 44] suffer from low abundance and overwhelming host contamination. In contrast, the circular structure of eccDNAs allows signal amplification via RCA, while effectively minimizing interference from linear cell-free DNA.

R patients exhibited distinct circulating eccDNA profiles compared to NR patients, notably with enrichment of eccDNAs derived from chr9 and an increased proportion of smaller-sized eccDNAs. The consistent enrichment of chr19-derived eccDNAs in both groups likely stems from preferential eccDNA biogenesis in high GC-content regions [45]; chr19, with the highest GC content (47.94%) among all human chromosomes [46], exemplifies this. The preferential production of eccDNAs from chr9 (GC content: 41.28%) [46] in R patients may also explain the slightly reduced GC peak value observed in this group.

A novel and notable finding was the identification of a previously undetected population of large eccDNAs (4.9–5.0 kb) in plasma. This breakthrough detection may be attributed to the time of RCA reaction was prolonged from 24 [40] to 60 h. These eccDNAs displayed a conserved structural pattern encompassing the full coding region (exons 1–8) and the first intron of *CKM*. Given this composition, they are unlikely to originate directly from simple genomic fragments, suggesting instead formation via: (1) fusion of multiple smaller eccDNAs, or (2) circularization following reverse transcription of spliced mRNA. These eccDNAs harboring the complete protein-coding *CKM* gene sequence have the potential to promote *CKM* amplification [47]. Overexpression of creatine kinase promotes tumor growth, particularly in CRC [48]. Thus, this eccDNA population represents a promising new candidate for CRC detection.

The circulating eccDNA profile in CRC patients was predominantly composed of sequences from intronic and intergenic regions, consistent with prior observations across various cancers [40]. However, we detected a greater number of exon-derived eccDNAs, likely due to the presence of the newly identified 4.9–5.0 kb eccDNAs, mainly composed of *CKM* exons. Additionally, R patients exhibited higher levels of promoter-derived eccDNAs than NR patients, particularly eccPromoter-CARD9. Functionally, eccPromoter-CARD9 promoted proliferation and migration in HCT116 cells and transcriptionally upregulated *DDIT3*, *ASNS*, and *CXCL8*. The elevated expression of the key transcription factor *DDIT3* is linked to reduced overall survival in CRC patients [26]. *ASNS* promotes the proliferation and metastasis of CRC cells [27]. Elevated *CXCL8* mediates anoikis resistance and correlates with poor CRC prognosis [28]. Therefore, the above results support the potential of eccPromoter-CARD9 as an oncogenic driver and a therapeutic target. The junctional sequences of eccPromoter-CARD9 offer potential therapeutic targets for selective removal via CRISPR/Cas9 editing while preserving genomic integrity [49]. 

We also identified elevated eccDNAs carrying the full-length hsa-mir-374c cluster in both tumor tissues and plasma of R patients. These molecules can drive oncogenic effects through enhanced proliferation and apoptotic resistance and mediate overexpression of MIR421 [20]. MIR421 has been well-characterized as an oncomiR involved in various malignancies, promoting metastasis via interacting with *CASP3* [50]. Furthermore, elevated MIR421 expression serves as a reliable non-invasive signal for CRC screening [51]. Thus, eccDNAs carry the full-length hsa-mir-374c cluster demonstrate potential as novel biomarkers for real-time monitoring of CRC recurrence. Superior to MIR421, these eccDNAs offer advantages in stability due to their resistance to RNase and exonuclease degradation. This renders them less demanding in terms of the environment and storage conditions, thus being more suitable for clinical testing.

Utilizing a classifier based on circulating eccGenes, we achieved robust discrimination between R and NR patients, with recurrence scores predictive of recurrence-free survival. These scores also increased with TNM stage and correlated positively with serum CA125 levels—an established independent prognostic marker in advanced CRC [52]—collectively affirming their utility in non-invasive risk stratification and guiding adjuvant therapy decisions. Following clarification of eccDNA’s role in recurrence prediction, the subsequent focus was extended to recurrence-related mortality to develop biomarkers for post-recurrence therapeutic stratification. Distinct circulating eccDNA profiles were identified between RS and RD patients. A panel of five eccGenes—eccCACNA1C-AS2, eccCROCC, eccMIR4297, eccTGFBR2, and eccTPO—was significantly elevated in RD patients. These genes carried by eccGenes contribute to cancer progression: *CACNA1C-AS2* functions as a tumor suppressor [53], *CR*OCC and *TPO* mediate cancer metastasis [54, 55], and *TGFBR2* promotes migration and invasion [56]. Classifiers built on these markers achieved high predictive performance for CRC mortality. Taken together, these circulating eccDNA-based models provide convenient and non-invasive tools for preoperative risk stratification, potentially accelerating clinical decision-making and surveillance.

Despite these encouraging results, several limitations still need to be addressed. First, this single-center study focused primarily on Chinese CRC patients, necessitating validation in multi-center and international cohorts. Second, the relatively small sample size underscores the need for further large-scale studies to confirm the generalizability and robustness of our prognostic models. Additionally, further analysis associating our prognostic models derived from peripheral blood with genomic alterations in tumor tissues will help uncover the biological functions of peripheral eccGene biomarkers and reinforce their specificity in CRC mutation subtypes.

## Conclusions

In summary, this study provides compelling evidence for the clinical and biological relevance of eccDNAs in recurrent CRC. We have established a comprehensive portrayal of circulating eccDNA abnormalities in CRC patients with recurrence and developed several effective non-invasive models for predicting both recurrence and mortality. These eccDNA-based classifier hold promise for improving risk stratification, facilitating detection of minimal residual diseases, and guiding personalized treatment decisions.

## Supplementary Information

Supplementary Material 1.

## Data Availability

Sequencing data could be downloaded from https://ngdc.cncb.ac.cn/omix/ (accession No. OMIX010503).
