# Supplementary material for: Circulating extrachromosomal circular DNA as a prognostic biomarker for colorectal cancer
Source: Cell Commun Signal. 2026 Feb 7;24:165. doi: 10.1186/s12964-026-02721-6 (PMC12977730; doi:10.1186/s12964-026-02721-6)
Supplement: Supplementary file 1 — Supplementary Material 1. [file 12964_2026_2721_MOESM1_ESM.docx]

**Supplementary Table 1.** Demographic and clinical characteristics of enrolled CRC patients. R, recurrence; NR, non-recurrence

| Patient Name | Gender | Age | Pathologic Diagnosis |
| --- | --- | --- | --- |
| NR1 | male | 78 | NR |
| NR2 | male | 75 | NR |
| NR3 | male | 55 | NR |
| NR4 | female | 69 | NR |
| NR5 | female | 47 | NR |
| NR6 | male | 49 | NR |
| NR7 | male | 71 | NR |
| NR8 | female | 53 | NR |
| NR9 | female | 81 | NR |
| NR10 | male | 68 | NR |
| NR11 | male | 54 | NR |
| NR12 | female | 70 | NR |
| NR13 | male | 46 | NR |
| NR14 | female | 71 | NR |
| NR15 | male | 72 | NR |
| NR16 | male | 66 | NR |
| NR17 | male | 71 | NR |
| NR18 | male | 64 | NR |
| NR19 | female | 64 | NR |
| NR20 | male | 71 | NR |
| NR21 | male | 54 | NR |
| NR22 | male | 80 | NR |
| NR23 | male | 63 | NR |
| NR24 | female | 61 | NR |
| NR25 | female | 63 | NR |
| NR26 | male | 66 | NR |
| NR27 | male | 57 | NR |
| NR28 | female | 54 | NR |
| NR29 | male | 61 | NR |
| NR30 | female | 51 | NR |
| NR31 | female | 85 | NR |
| NR32 | male | 88 | NR |
| NR33 | female | 60 | NR |
| NR34 | female | 62 | NR |
| NR35 | female | 66 | NR |
| NR36 | male | 60 | NR |
| NR37 | female | 86 | NR |
| NR38 | male | 62 | NR |
| NR39 | male | 57 | NR |
| NR40 | female | 52 | NR |
| NR41 | male | 66 | NR |
| NR42 | female | 55 | NR |
| NR43 | female | 62 | NR |
| NR44 | male | 64 | NR |
| NR45 | female | 54 | NR |
| NR46 | female | 56 | NR |
| NR47 | female | 59 | NR |
| NR48 | female | 75 | NR |
| NR49 | female | 59 | NR |
| NR50 | male | 55 | NR |
| NR51 | male | 60 | NR |
| NR52 | female | 67 | NR |
| NR53 | female | 70 | NR |
| NR54 | male | 74 | NR |
| NR55 | male | 62 | NR |
| NR56 | female | 75 | NR |
| NR57 | male | 71 | NR |
| NR58 | male | 68 | NR |
| NR59 | female | 65 | NR |
| NR60 | male | 62 | NR |
| NR61 | male | 41 | NR |
| NR62 | male | 64 | NR |
| NR63 | male | 54 | NR |
| NR64 | male | 63 | NR |
| NR65 | male | 60 | NR |
| NR66 | male | 59 | NR |
| NR67 | male | 61 | NR |
| NR68 | male | 62 | NR |
| NR69 | female | 64 | NR |
| NR70 | female | 67 | NR |
| NR71 | female | 60 | NR |
| NR72 | male | 79 | NR |
| NR73 | female | 79 | NR |
| NR74 | male | 62 | NR |
| NR75 | female | 62 | NR |
| NR76 | female | 62 | NR |
| NR77 | female | 60 | NR |
| NR78 | female | 75 | NR |
| NR79 | male | 69 | NR |
| NR80 | female | 84 | NR |
| NR81 | male | 73 | NR |
| NR82 | male | 59 | NR |
| NR83 | male | 64 | NR |
| NR84 | male | 63 | NR |
| NR85 | male | 53 | NR |
| NR86 | male | 66 | NR |
| NR87 | male | 54 | NR |
| NR88 | male | 70 | NR |
| NR89 | male | 80 | NR |
| NR90 | male | 65 | NR |
| NR91 | male | 60 | NR |
| NR92 | male | 79 | NR |
| NR93 | female | 40 | NR |
| NR94 | male | 49 | NR |
| NR95 | female | 65 | NR |
| NR96 | female | 47 | NR |
| NR97 | male | 61 | NR |
| NR98 | male | 71 | NR |
| NR99 | male | 60 | NR |
| NR100 | female | 66 | NR |
| NR101 | female | 46 | NR |
| NR102 | male | 70 | NR |
| NR103 | male | 88 | NR |
| NR104 | male | 72 | NR |
| NR105 | male | 77 | NR |
| NR106 | male | 84 | NR |
| NR107 | male | 59 | NR |
| NR108 | male | 55 | NR |
| NR109 | female | 62 | NR |
| NR110 | female | 48 | NR |
| NR111 | female | 86 | NR |
| NR112 | female | 66 | NR |
| NR113 | male | 73 | NR |
| NR114 | female | 80 | NR |
| NR115 | male | 84 | NR |
| NR116 | male | 59 | NR |
| NR117 | female | 64 | NR |
| NR118 | male | 81 | NR |
| NR119 | male | 56 | NR |
| NR120 | female | 71 | NR |
| NR121 | female | 81 | NR |
| NR122 | male | 79 | NR |
| NR123 | male | 65 | NR |
| NR124 | female | 49 | NR |
| NR125 | male | 72 | NR |
| NR126 | male | 82 | NR |
| NR127 | male | 66 | NR |
| NR128 | female | 59 | NR |
| NR129 | male | 53 | NR |
| NR130 | male | 80 | NR |
| NR131 | male | 53 | NR |
| NR132 | male | 73 | NR |
| NR133 | male | 79 | NR |
| R1 | male | 83 | R |
| R2 | male | 56 | R |
| R3 | male | 77 | R |
| R4 | male | 77 | R |
| R5 | male | 66 | R |
| R6 | female | 69 | R |
| R7 | male | 40 | R |
| R8 | male | 53 | R |
| R9 | female | 59 | R |
| R10 | female | 79 | R |
| R11 | female | 54 | R |
| R12 | female | 51 | R |
| R13 | male | 82 | R |
| R14 | female | 68 | R |
| R15 | female | 78 | R |
| R16 | female | 72 | R |
| R17 | female | 57 | R |
| R18 | male | 60 | R |
| R19 | male | 42 | R |
| R20 | female | 47 | R |

**Supplementary Table 2.** Baseline characteristic of CRC patients. Values are median (interquartile range) or n (%).

|  | Recurrence  (N = 20) | Non-recurrence  (N = 133) | *P* value |
| --- | --- | --- | --- |
| Male | 10 (50.0%) | 81 (60.9%) | 0.464 |
| Age, year | 63.00 (53.75–77.00) | 64.00 (59.00–72.00) | 0.527 |
| Previous history |  |  |  |
| Smoking | 5 (25.0%) | 39 (29.3%) | 0.796 |
| Alcohol | 4 (20.0%) | 35 (26.3%) | 0.784 |
| Other cancers | 1 (5.0%) | 10 (7.5%) | 1.000 |
| Family history | 4 (20.0%) | 23 (17.3%) | 0.756 |
| Histological subtype |  |  |  |
| Adenocarcinoma | 19 (95.0%) | 126 (94.7%) | 1.000 |
| Mucinous adenocarcinoma | 1 (5.0%) | 7 (5.3%) |  |
| Pathological stage |  |  |  |
| TNM I | 0 (0.0%) | 26 (19.5%) | < 0.001 |
| TNM II | 4 (20.0%) | 59 (44.4%) |  |
| TNM III | 8 (40.0%) | 42 (31.6%) |  |
| TNM IV | 8 (40.0%) | 6 (4.5%) |  |

**Supplementary Table 3.** Primer sequences.

Primers for eccDNA (chr9:136,374,457-136,374,791) synthesis

| Primer Name | Primer Sequences |
| --- | --- |
| CARD9_F | GTTGAACTGAGTTGCCACCC |
| CARD9_R | TCCGCGTTTTAAACGCTG |
| Overlapping fragment_F | CAGCGTTTAAAACGCGGAGTTGAACTGAGTTGCCACCC |
| Overlapping fragment_R | GGGTGGCAACTCAGTTCAACTCCGCGTTTTAAACGCTG |
| Complementary dsDNA1_F | 5’P-GTTGAACTGAGTTGCCACCC |
| Complementary dsDNA1_R | 5’P-TCCGCGTTTTAAACGCTG |
| Complementary dsDNA2_F | 5’P-GAGCTTGCCTAACCGTTCTC |
| Complementary dsDNA2_R | 5’P-CACACCAGCTTCCTTGCCT |

Primers for RT-qPCR validation

| Primer Name | Primer Sequences |
| --- | --- |
| ACTB_F | CACCATTGGCAATGAGCGGTTC |
| ACTB_R | AGGTCTTTGCGGATGTCCACGT |
| DDIT3_F | GGTATGAGGACCTGCAAGAGGT |
| DDIT3_R | CTTGTGACCTCTGCTGGTTCTG |
| ASNS_F | CTGTGAAGAACAACCTCAGGATC |
| ASNS_R | AACAGAGTGGCAGCAACCAAGC |
| CXCL8_F | GAGAGTGATTGAGAGTGGACCAC |
| CXCL8_R | CACAACCCTCTGCACCCAGTTT |


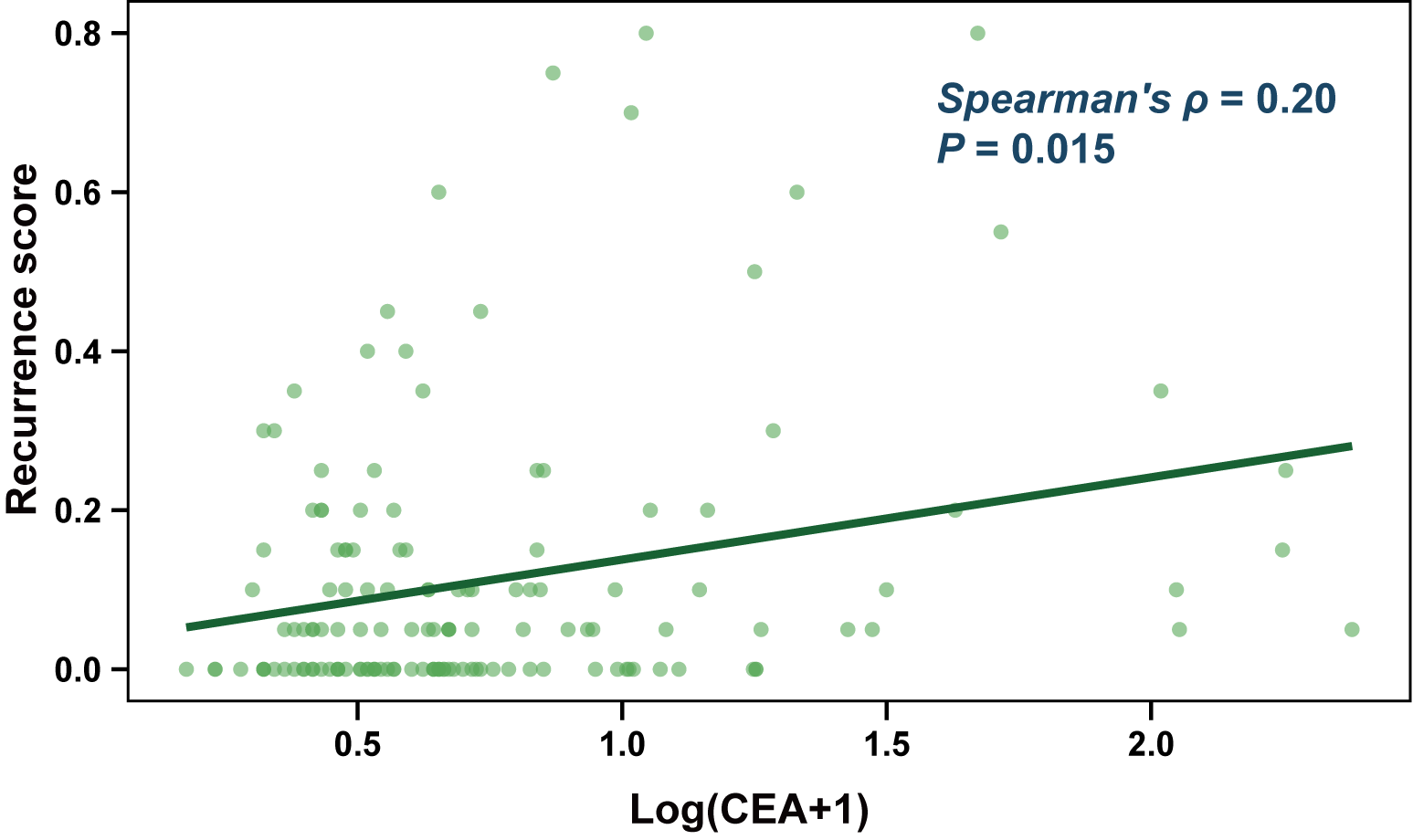


Supplementary Figure 1. Spearman correlation between recurrence scores and serum levels of carcinoembryonic antigen (CEA).
